# Type 1 Achilles tendon rupture caused by grooming trauma in a young dog

**Published:** 2014-05-02

**Authors:** M. Isaka, M. Befu, N. Matsubara, M. Ishikawa, H. Aono, S. Namba

**Affiliations:** 1 *Marble Veterinary Medical Center, 4-1-6 Ishikawa, Fujisawa, Kanagawa, Japan 252-0815*; 2 *Aono Pet Clinic, 31-2 Fukawa, Odawara, Kanagawa, Japan 250-0052*

**Keywords:** Achilles tendon, Dog, Rupture

## Abstract

Achilles tendon rupture is uncommon in small animal practice. A 9-month-old, female, mixed breed dog (weighing 2.2kg) was referred to our hospital with a primary complaint of right hind limb lameness. Complete right Achilles tendon rupture was diagnosed by physical examination and radiography. The tendon was surgically repaired the next day by using a three-loop and single near-far-far-near suture methods. Complete healing was achieved by 97 days post-surgery. This report describes the surgical technique used for complete Achilles tendon rupture repair in a young dog.

## Introduction

Canine Achilles tendon rupture is a rare orthopedic condition. It occurs primarily in large breed dogs and is usually caused by trauma. Diagnosis is based on radiography, ultrasonography, and MRI, but physical examination and posture/gait analysis are also useful indicators. Surgical repair is the sole therapy. The ruptured Achilles tendon is sutured and ankle joint is stabilized for several weeks.

Achilles tendon rupture is classified into the following types based on severity: (1) complete, (2a) muscle-tendon rupture, (2b) complete tendon rupture with intact paratenon, (2c) gastrocnemius injury or complete superficial digital flexor tendon rupture, and (3) chronic tendonitis or calcific tendinitis (Corr *et al.*, 2010). Type I rupture is the most common; the tendon is reconstructed with a three-loop suture method, locking loop suture method, and arthrodesis by external fixation or kirschner pin for an extended period. In the present case study, we describe surgical repair of type I Achilles tendon rupture in a puppy that was caused by a grooming injury, using a three-loop and near-far-far-near methods.

### Case Details

**Fig. 1:**
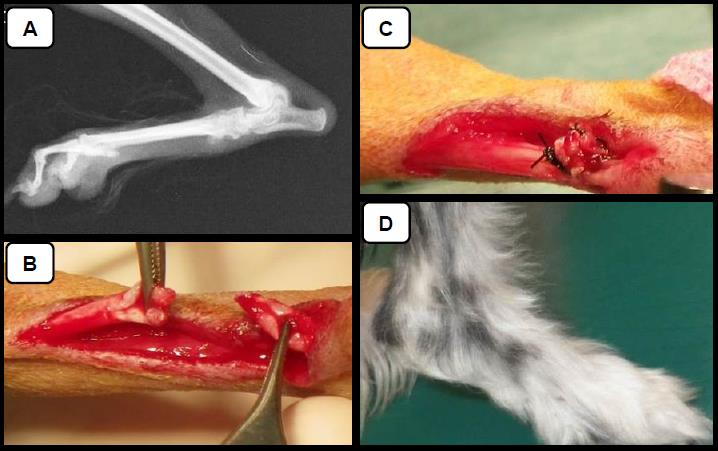
(**A**) Radiography of right limb at the diagnosis. (**B**) Type I Achilles tendon rupture was confirmed intra-operatively. (**C**) The tendon was repaired with a three-loop suture method and a single near-far-far-near suture method with 3-0 non-absorbable sutures. (**D**) At 97 days, the limb was 100% weight bearing and no limping was noted.

A female mixed breed dog (Toy poodle and Maltese), aged 9 months, and weighting (2.2kg) was evaluated. The primary veterinarian diagnosed a right Achilles tendon rupture caused by a grooming injury and referred the patient to our hospital. Physical examination and radiography confirmed a right Achilles tendon rupture (Fig. 1A).

The next day, we performed surgery under general anesthesia. Robenacoxib (2mg/kg) was subcutaneously administered prior to anesthesia, and anesthesia was induced with intravenous propofol (8mg/kg). The trachea was intubated and anesthesia was maintained with inhaled isoflurane. A standard approach was used to expose the joint and a type I Achilles tendon rupture was confirmed intra-operatively (Fig. 1B).

The tendon was repaired with a three-loop suture method and a single near-far-far-near suture method with 3-0 non-absorbable sutures (Monosof) (Fig. 1C).

A Robert Jones bandage was applied to the limb postoperatively to limit the range of motion to 110°. The patient was hospitalized 4 days following surgery; a cold compression was applied to the incision for 10 min twice daily and ampicillin (20mg/kg) was administered subcutaneously followed by oral amoxicillin (20mg/kg) until suture removal.

The Robert Jones bandage was removed 32 days postoperatively. By this time, the limb was capable of partial weight bearing and the patient was able to limp and walk; hence the in-home exercise limitation was lifted. It was difficult for the owner to visit our hospital, and therefore, we prescribed water rehabilitation (water level, 3cm under the stifle joint; twice a week; 15 min per session).

At 65 days postoperatively limping was diminished, and the limb was 80-90% weight bearing, therefore, water rehabilitation was reduced to one 15-min session per week. At 97 days, the limb was 100% weight bearing and no limping was noted; thus, therapy was discontinued (Fig. 1D).

## Discussion

In a retrospective study of 45 cases, canine Achilles tendon rupture occurred in middle or large breed dogs such as Doberman Pinscher, Labrador Retriever, Border Collie. Most cases were acute onset (66.7%); the injury severity included, complete tendon rupture (26.7%), superficial digital flexor tendon rupture (22.2%), and gastrocnemius rupture (20%).

Most of the Achilles tendon ruptures occurred at the tendon-bone junction (60%), and a smaller percentage occurred at the muscle-tendon junction (20%) and within the tendon itself (13.3%) (Corr *et al.*, 2010). Plantigrade gait (walking on the soles of the feet) was not specifically described in the retrospective study, but our patient did exhibit plantigrade gait.

Diagnosis was very simple in our patient, which clearly exhibited the characteristic clinical signs of Achilles tendon rupture. In general, the only therapy is surgical repair divided into suture methods, which include locking-loop and three-loop methods, and stabilization, which include calcaneotibial immobilization with implanted screws or casting.

The surgical result depends on whether the wound is opened or closed, time elapsed since injury, and rupture severity; surgical technique does not affect the surgical outcome. In the previously mentioned retrospective study, the complication rate was 16 of 45 cases (35%); 10 cases had mild complications, and 6 cases had severe complications.

The complication rate increased and the long-term prognosis worsened when the Achilles tendon was ruptured. In the present case, although a complete rupture was diagnosed, the owner was very satisfied with the lack of complications, and good surgical results.

In feline Achilles tendon rupture, a study evaluating treatment with external skeletal fixators had a 33% complication rate that was attributed to the method of external coaptation. By contrast, a splint immobilization did not cause any complications.

In feline type I and II Achilles tendon rupture, there was no difference in the long-term clinical outcomes of conservative (79%) and surgical (84%) repairs (Cervi *et al.*, 2010). While our patient’s weight was similar to that of a cat, we performed a three-loop and single near-far-far-near method with Robert Jones bandage at the owner’s request.

In dogs, an alternative surgical technique is augmentation of the primary Achilles tendon repair with a three-loop suture method and a semitendinosus muscle flap in dogs. Further investigation of the technique’s long-term clinical outcome is needed (Baltzer and Rist, 2009).

We applied a Robert-Jones bandage for 32 days because post-operative immobilization is an essential part of therapy (Morshead and Leeds, 1984). Achilles tendon immobilization can be achieved with either a Robert Jones bandage or Kirschner-Ehmer apparatus. Postoperative Kirschner-Ehmer apparatus has a good result without tenorraphy failure in the dog (Morshead and Leeds, 1984). However, a Robert Jones bandage is noninvasive and may be more practical in a small breed dog, such as our patient.

Surgical treatment of Achilles tendon rupture in dogs is generally associated with a favorable outcome, although the recovery time to full function is relatively long at 20.2 weeks. Dogs with injuries of less than 21 days duration may have a better functional outcome. When comparing external fixator application to splint or cast management, the initial tibiotarsal immobilization method does not significantly affect the complication rate, duration of immobilization, recovery time, or functional outcome (Nielsen and Pluhar, 2006). In our case, recovery time was relative shorter than reported by Nielsen and Pluhar (2006); the good surgical results may reflect the acute onset, rapid diagnosis, and immediate surgery.

Suture techniques for tendon anastomosis vary and include: Bunnel-Mayer, Mason- Allen, simple interrupted, locking loop or modified Kessler, double locking loop, three-loop pulley, Krackow, continuous cruciate, and near-far-far-near (Fahie, 2005). Repair with a locking loop suture and own suture technique gives good results (Adamiak and Holak, 2005), the 3-loop pulley pattern is more resistant to gap formation during tensile loading, and is quicker to place, than 2 locking-loop sutures.

In addition, gap formation can significantly delay tendon healing. Tendon repairs with a gap >3mm have an increased risk of rupture during the first 6 weeks postoperatively (Moores *et al.*, 2004a, b). Thus, the 3-loop pulley pattern is superior to the 2 locking-loop pattern. We used a 3-loop pulley pattern with near-far-far-near pattern and did not create a gap, leading to a good surgical result consistent with the previous study. Comparison of the three-loop and locking loop methods in vivo and clinically have been reported. A modified 3-loop pulley pattern is biomechanically superior to a locking-loop pattern for reattachment of the canine gastrocnemius tendon to bone and may be suitable for clinical use.

In our case, a 3-loop pulley pattern and near-far-far-near suture method eliminated the tendon gap, leading to full healing.

Our surgical result suggests that these suture techniques combined with a Robert-Jones bandage could result in a good prognosis in small breed dogs without requiring a cast, external fixator, or stabilization using screws. Finally, we advise that the veterinarian should counsel pet owners on ways to avoid grooming injuries.
